# Durable response to the combination of pembrolizumab and nab-paclitaxel in a metastatic extrahepatic cholangiocarcinoma: A case report and literature review

**DOI:** 10.3389/fphar.2022.1037646

**Published:** 2022-11-28

**Authors:** Sirui Tan, Jing Yu, Qiyue Huang, Nan Zhou, Xianze Xiong, Hongfeng Gou

**Affiliations:** ^1^ Department of Medical Oncology, Gastric Cancer Center, West China Hospital, Sichuan University, Chengdu, Sichuan, China; ^2^ Department of Bile Duct Surgery, West China Hospital, Sichuan University, Chengdu, China

**Keywords:** cholangiocarcinoma, immunotherapy, nab-paclitaxel, pembrolizumab, tumor microenvironment

## Abstract

**Background:** Cholangiocarcinoma (CCA) is a highly aggressive malignant tumor with poor overall survival. Although the first-line standard chemotherapy (gemcitabine plus cisplatin) combined with immunotherapy has yielded positive results with survival prolongation, the efficacy remains unsatisfactory, and new treatment modalities need to be explored.

**Case presentation:** We report the case of a patient with metastatic extrahepatic CCA who achieved a durable response and good tolerance to the combination treatment of pembrolizumab and nab-paclitaxel following progression on gemcitabine plus capecitabine chemotherapy. The tumor samples of the patient revealed low TMB, MSS, negative PD-L1 expression, and negative CD8^+^ TIL expression. This patient was treated with 3 cycles of pembrolizumab plus nab-paclitaxel and cisplatin, followed by 5 cycles of pembrolizumab plus nab-paclitaxel. Finally, 10 cycles of pembrolizumab monotherapy were administered. The patient survived for over 27 months after the initiation of combined therapy and was still in continuous remission at the last follow-up.

**Conclusion:** As far as we know, this is the first report that pembrolizumab plus nab-paclitaxel successfully treated a patient with advanced CCA. This combination therapy might be a potential treatment option for patients with cholangiocarcinoma, and further clinical trials are needed to explore the outcomes.

## Background

Cholangiocarcinoma (CCA) originates from the bile duct cells and accounts for 3% of digestive system tumors (Rizvi and Gores, 2013). Its morbidity and mortality have risen in recent years (Khan et al., 2019). According to anatomical location, Cholangiocarcinoma is categorized as intrahepatic cholangiocarcinoma (iCCA) and extrahepatic cholangiocarcinoma (eCCA). In addition, eCCA can be divided into perihilar CCA (pCCA) and distal CCA (dCCA) (Rizvi and Gores, 2013). Cholangiocarcinoma is highly aggressive, and surgery is still the primary treatment option. However, more than two-thirds of patients are unsuitable for surgery at the initial diagnosis (Jarnagin et al., 2001). Patients who have undergone radical surgery have a high rate of recurrence and metastasis (Jung et al., 2012). The prognosis of metastatic cholangiocarcinoma is extremely poor, with a 5-year overall survival (OS) rate of about 10% (Everhart and Ruhl, 2009; Tyson and El-Serag, 2011).

For patients with advanced cholangiocarcinoma, systemic chemotherapy is the backbone of palliative care, and commonly used agents include gemcitabine, platinum, and fluoropyrimidines. The United Kingdom ABC-02 study established cisplatin and gemcitabine (CisGem) as the reference first-line regimen, with an improved OS (11.7 vs. 8.1 months) compared with gemcitabine monotherapy (Valle et al., 2010). Recently, in a promising single-arm phase II study, the combination of CisGem plus nab-paclitaxel provided a response rate (RR) of 45% and a median OS of 19.2 months in chemotherapy-naïve patients (Shroff et al., 2019). The median OS with first-line reference doublet is less than one year, and the high incidence of adverse effects of intensified triple-agent cannot be ignored. In the second-line setting, the only phase III trial is the ABC-06 study which assessed the benefit of the regimen of mFOLFOX (oxaliplatin, leucovorin, 5-fluorouracil) compared with active symptomatic control in CisGem-refractory patients. Although the median OS of patients receiving mFOLFOX was statistically significant, the OS improvement was modest (6.2 vs. 5.3 months) (Lamarca et al., 2021). The limited survival benefit of current chemotherapy options highlights the need to develop more effective therapeutic options. The molecular heterogeneities in CCA have been identified with the emergence of next-generation sequencing. The patients carrying fibroblast growth factor receptor 2 (FGFR2) fusion/rearrangement, isocitrate dehydrogenase (IDH) mutation, and neurotrophin receptor tyrosine kinase (NTRK) genes fusion may benefit from the corresponding targeted therapy (Mazzaferro et al., 2019; Abou-Alfa et al., 2020; Bekaii-Saab et al., 2020; Doebele et al., 2020; Lamarca et al., 2020; Rizzo et al., 2021b). However, these actionable molecular alterations usually occur in intrahepatic cholangiocarcinoma (Abou-Alfa et al., 2020; Montal et al., 2020; Rizzo et al., 2020).

In recent years, immune checkpoint inhibitors (ICIs) have shown outstanding efficacy in pan-tumors such as malignant melanoma, lung cancer, urothelial cancer, and liver cancer (Balar et al., 2017; Eggermont et al., 2018; Xu et al., 2018; Mok et al., 2019). The results of early clinical trials of ICIs monotherapy (pembrolizumab, nivolumab, and durvalumab) in unselected patients with biliary tract cancer (BTC) provided limited activity, with response rate (RR) ranging between 3% and 13% (Bang et al., 2019; Ueno et al., 2019; Kim et al., 2020; Rizzo et al., 2021a). A number of different ICIs combinations are under investigation, and the combination of ICIs and chemotherapy (chemoimmunotherapy) has shown promising anti-tumor efficacy. Notably, data from the placebo-controlled, phase III TOPAZ-1 trial demonstrated that durvalumab plus CisGem signficantly improved survival outcomes (12.8 vs. 11.5 months) compared to CisGem alone as a first-line treatment in advanced BTC (Oh et al., 2022). Patients receiving the chemoimmunotherapy also had an improved progression-free survival (7.2 vs. 5.7 months) and RR (26.7% vs. 18.7%). This combination is currently considered a new standard of care in first-line advanced BTC. The first-line, placebo-controlled phase 3 study of pembrolizumab (NCT04003636) in combination with CisGem is underway. Most chemoimmunotherapy trials in advanced BTC applied CisGem or oxaliplatin-based regimens as the chemotherapy backbone. However, the net benefit was not satisfying, and new combinations still need to be investigated. Here, for the first time, we report a patient with metastatic extrahepatic cholangiocarcinoma who had a durable response and good tolerance to pembrolizumab combined with nanoparticle albumin-bound (nab)-paclitaxel in a background of low tumor mutation burden (TMB), microsatellite stable (MSS), negative PD- L1 expression and negative CD8^+^ tumor-infiltrating lymphocyte (TIL) expression in the tumor microenvironment (TME).

## Case presentation

On 15 April 2019, a 66-year-old man was admitted to the West China Hospital with pain in his lower back and jaundice of the skin and sclera. The patient has no other medical, family, or psychosocial history.

**FIGURE 1:**
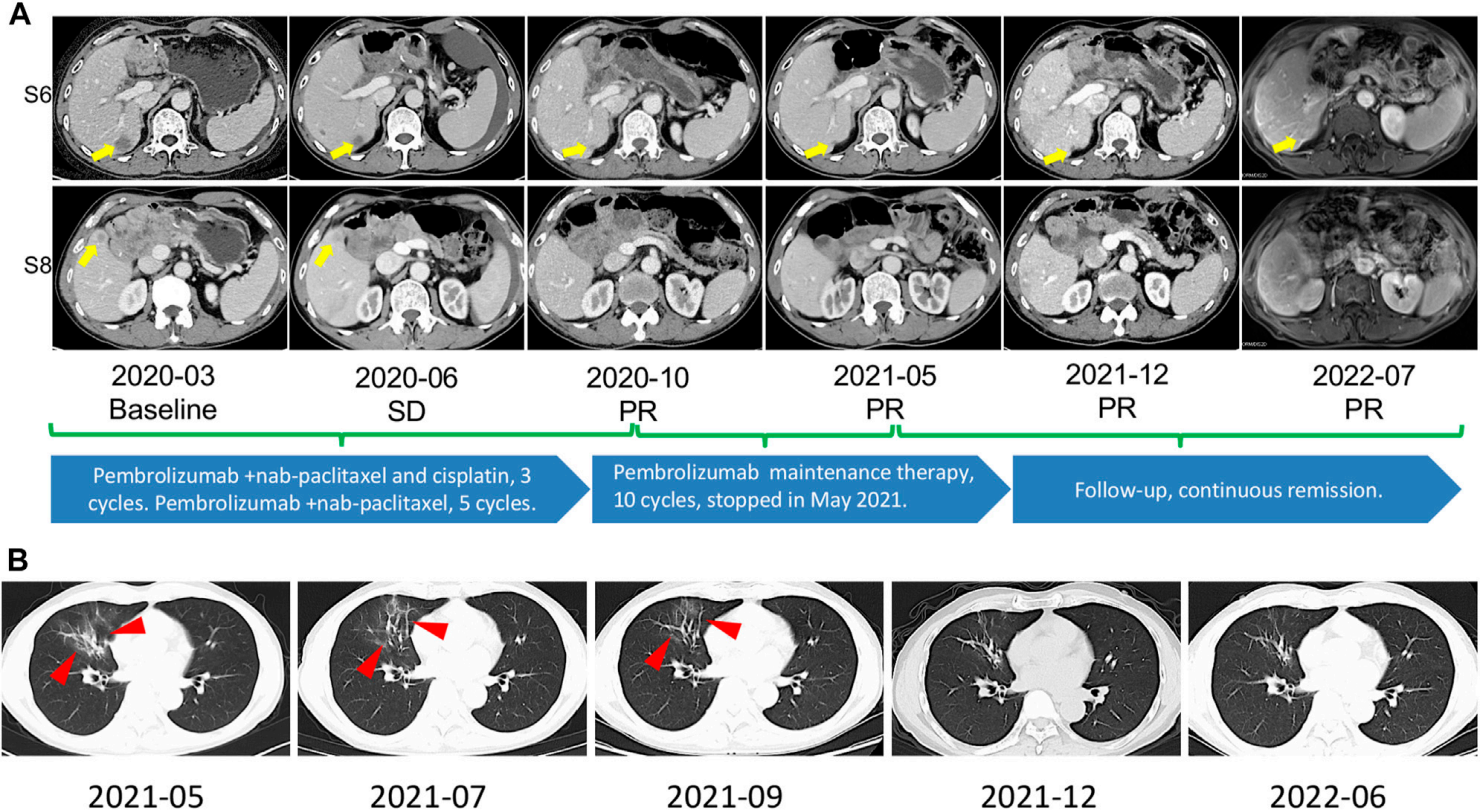
**(A)** The timeline and representative CT and MRI images. CT of March 2020, yellow arrow points toward intrahepatic lesions in S6/S8 at tumor metastasis. CT of June 2020 revealed stable disease was observed after 2 cycles of nab-paclitaxel/cisplatin plus pembrolizumab. CT of October 2020, partial remission was observed after 3 cycles of nab-paclitaxel/cisplatin plus pembrolizumab and 5 cycles of nab-paclitaxel plus pembrolizumab. CT of May 2021, continuous partial remission was observed after 10 cycles of pembrolizumab maintenance therapy. In July 2022, MRI showed durable partial remission. S6, Segment 6; S8, Segment 8; SD, stable disease; PR, partial remission. **(B)** Image changes during prednisolone treatment after the onset of CIP. CIP was significantly relieved, as seen in the images. The red arrow points to the part where CIP occurred. CIP, checkpoint inhibitor pneumonitis.

**FIGURE 2:**
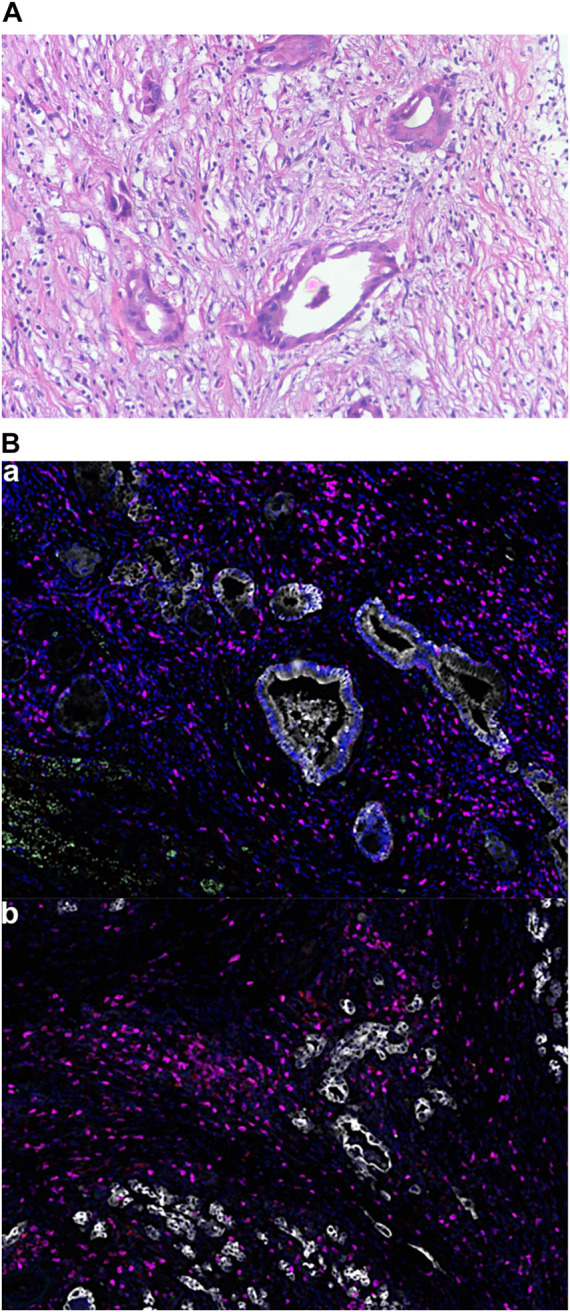
Tumor pathological findings. **(A)** Hematoxylin and eosin staining of the patient’s tumor. Magnification, x200; **(B)** Multiple immunofluorescence of the TME: (a): Green for PD-1; Yellow for PD-L1; Pink for CD8; Cyan for CD68; Red for CD163. Magnification, x200. (b): Pink for CD3; Red for CD4; Green for CD20; Cyan for CD56; Yellow for FoxP3. Magnification, x200.

Computed tomography (CT) scan showed dilatation of the common bile duct, with thickened lower duct wall and mild enhancement. A radical pancreaticoduodenectomy was performed on 22 April 2019. Postoperative pathology revealed a moderately-poorly differentiated adenocarcinoma, with invasion to the common bile duct wall, duodenal wall and pancreatic parenchyma. Nerve invasion and intravascular emboli were also observed. There were no regional lymph node metastases. Immunohistochemical (IHC) analysis showed CK7(+), CK19(+), MUC-1 (+), MUC-2 (-), DPC-4 (+/-), CK (focal+), CDX-2 (-). The patient was diagnosed as stage IIIB-T4N0M0. Chest and abdominal CT were performed two months after surgery, showing no signs of tumor recurrence or metastasis. A total of 8 cycles of gemcitabine plus capecitabine (GEMCAP) as adjuvant chemotherapy was performed from 22 June 2019, to 18 December 2019. On 27 March 2020, abdominal CT showed multiple liver metastases with a 22 mm × 27 mm mass forming lesion in segment 6 (S6) and a 9 mm × 13.5 mm mass forming lesion in S8 (Figure 1A). Meanwhile, laboratory examinations showed that the patient’s carcinoembryonic antigen (CEA) was 7.69 ng/ml (normal level < 3.4 ng/ml), and the carbohydrate antigen 19–9 (CA 19–9) was 33.10 U/ml (normal level < 22 U/ml). Subsequently, the patient underwent next-generation sequencing (NGS) on surgically resected specimens, and the results showed KRAS and TP53 mutations, TMB of 1 mutation/megabase, and MSS. Multiple immunofluorescence was used to detect TME (Figure 2), indicating that PD-L1 expression was negative (TPS< 1%, CPS< 1) and CD8^+^ TIL expression was quantified at 0.21% of the tumor parenchyma and 1.15% of the stroma (Table 1). Based on the results of Gainor et al. (2016), the patient was judged “negative” for CD8+TIL expression.

**TABLE 1:** Cell composition of tumor immune microenvironment.

Cellular components		Tumor parenchyma		Tumor stroma	
		Number/mm^2^	%	Number/mm^2^	%
T cell related indicators	CD3	227	3.69	790	14.03
	CD4	15	0.25	77	1.37
	CD8	17	0.21	65	1.15
	FoxP3	15	0.24	49	0.88
	PD-1^+^CD8^+^	0	0.00	0	0.00
	CD4^+^FoxP3^+^	1	0.02	6	0.10
Macrophage related indicators	CD68^+^CD163^+^	0	0.00	1	0.02
	CD68^+^CD163^−^	36	0.44	166	2.94
	PD-L1^+^CD68^+^	1	0.01	4	0.06
NK cell related indicators	CD56bright	7	0.11	43	0.77
	CD56dim	72	1.17	128	2.27
B cell related indicators	CD20	14	0.22	82	1.45

**FIGURE 3:**
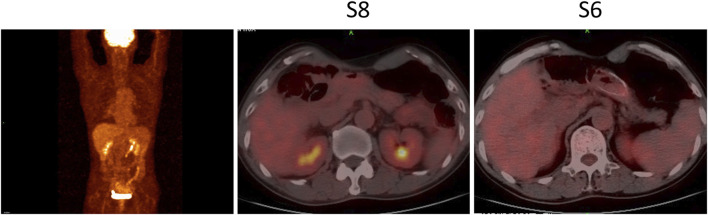
PET-CT in August 2020 showed disappeared hypermetabolic activities in the hepatic lesions and no clear sign of tumor recurrence and metastasis throughout the body. S6, Segment 6; S8, Segment 8.

**FIGURE 4:**
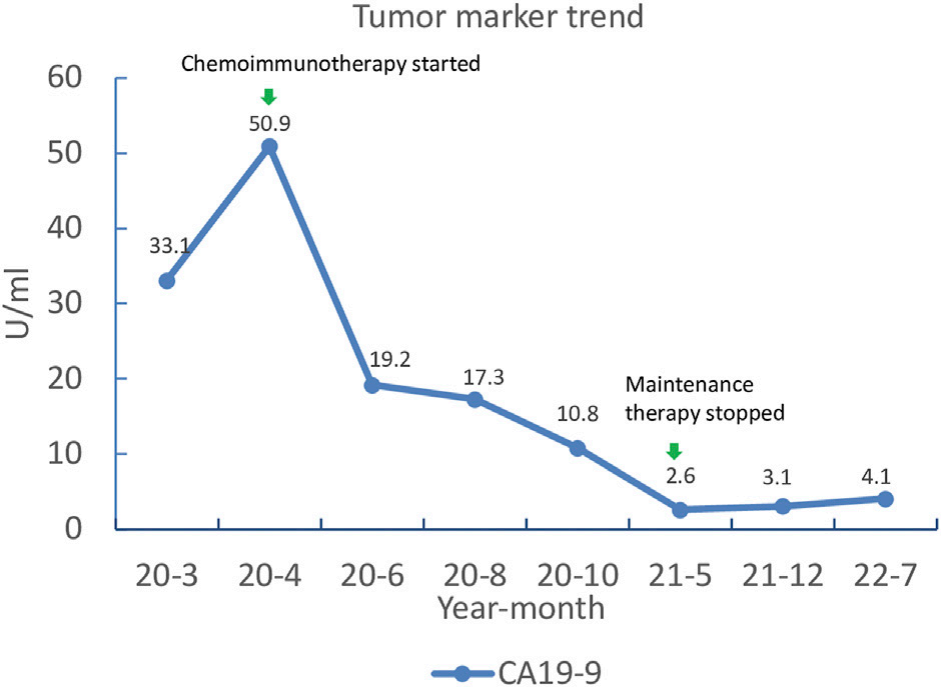
The response of tumor marker to chemoimmunotherapy. CA19–9, carbohydrate antigen 19–9.

Four months after adjuvant chemotherapy, the patient developed metastasis, and the GEMCAP regimen was considered the first-line treatment. Due to the limited benefit of the standard second-line chemotherapy (FOLFOX), a switch to second-line systemic therapy with nab-paclitaxel and cisplatin (AP) plus pembrolizumab was recommended by our tumor conference. Before treatment, written informed consent was obtained from the patient who presented Eastern Cooperative Oncology Group performance score (ECOG) of zero. In April 2020, the treatment with pembrolizumab plus AP was initiated. The first radiological imaging in June 2020 showed a stable disease after two cycles (Figure 1A). Due to grade 3 vomiting and grade 2 nausea (CTCAE5.0), cisplatin was discontinued after three cycles. Five cycles of pembrolizumab plus nab-paclitaxel were performed from July to October 2020. Although the positron emission tomography-computed tomography (PET-CT) in August 2020 showed no hypermetabolic activities in the hepatic lesions and no clear sign of tumor recurrence or metastasis throughout the body (Figure 3), the CT scan in October 2020 indicated a partial response (PR) (Figure 1A **)**, The patient’s CEA and CA 19–9 levels also returned to the normal range (Figure 4). The most common adverse events were hematologic toxicities (grade 2 leukopenia, grade 2 neutropenia and grade 1 anemia) during chemoimmunotherapy.

Subsequent ten cycles of pembrolizumab monotherapy (the maintenance therapy) were administered and stopped in May 2021, when grade 2 checkpoint inhibitor pneumonitis (CIP) was observed (Figure 1B). The prednisolone (initial dose at 1 mg/kg/day–2 mg/kg/day) was administered, and the dose was reduced by 5 mg per week. The final therapy was glucocorticosteroid treatment, which started in May 2021 and lasted for six weeks, and the patient did not receive any anti-tumor treatment since then. The patient survived for over 27 months after the chemoimmunotherapy and was still in continuous remission at the last follow-up in July 2022 (Figure 1A **)**.

## Discussion

Here, we report on the efficacy and tolerability of a combination of pembrolizumab plus nab-paclitaxel as a second-line therapy after GEMCAP regimen progression in a patient with low TMB, MSS, negative PD-L1 expression, and negative CD8^+^ TIL expression extrahepatic cholangiocarcinoma.

Recent evidence suggests that cytotoxic agents work synergistically with immunotherapy by boosting anti-tumor immunity. Cytotoxic agents majoring modulate the TME by inducing immunogenic tumor cell death and inhibiting mechanisms utilized by tumor cells for immune evasion (Galluzzi et al., 2015). The TOPAZ-1 phase III trial has shown a benefit for chemoimmunotherapy (durvalumab plus CisGem) compared to chemotherapy alone. Although the survival benefits are limited, the survival curves indicated that 24.9% of patients are still alive after two years and showed unprecedented durable responses that chemotherapy alone couldn’t reach (Oh et al., 2022). Based on this encouraging data, many trials comparing the effects of ICI alone or in combination with chemotherapy, such as KEYNOTE-966 (pembrolizumab + CisGem), IMBRAVE 151 (atezolizumab + CisGem), M7824 (bintrafuspare + CisGem), are ongoing. Most trials applied chemoimmunotherapy as the first-line treatment, with CisGem regimen chemotherapy backbone. In this case, the patient was treated with nab-paclitaxel-based chemoimmunotherapy as second-line therapy and achieved a long-lasting response. It is worth mentioning that our patient started with doublet chemotherapy in the second line of treatment but experienced unacceptable adverse effects and declining performance status. Therefore, single-agent chemotherapy combined with immunotherapy might be a good strategy from the perspective of patient tolerance in the second-line setting. The anti-tumor activity of nab-paclitaxel plus immunotherapy has been validated, with acceptable toxicity in backline treatment in other tumors (Schmid et al., 2018; Giannatempo et al., 2020; Wang et al., 2022). Nab-paclitaxel has been widely explored in patients with BTC in previous clinical trials (Sahai et al., 2018; Shroff et al., 2019; Woodford et al., 2021 June 28), while the efficacy of the combination of nab-paclitaxel plus immunotherapy as the second-line treatment in BTC patients has not been studied. Notably, nab-paclitaxel has a synergistic effect in combination with ICIs due to its special nanoparticle carrier (Martin et al., 2020). The underlying mechanisms are associated with enhancing antigen-presenting cells (APCs) antigen presentation ability, affecting the tumor immune microenvironment, and promoting T lymphocyte activation to kill tumor cells (Martin et al., 2020).

Selecting people who can benefit from immunotherapy remains one of the biggest problems. MSI-H/dMMR, high TMB, PD-L1 positive expression, and high immune cell infiltration are currently considered predictive biomarkers of response to immunotherapy or its combination with chemotherapy (Zhao et al., 2019). Previous studies have demonstrated great clinical activity of ICIs in BTC patients with microsatellite instability-high (MSI-H) or mismatch repair deficiency (dMMR) (Le et al., 2017). However, the incidence of dMMR/MSI-H BTC is less than 5% (Winkelmann et al., 2018; Goeppert et al., 2019). High TMB has been only reported in 5.9% of BTC patients, and the median TMB of BTC is about two mutations/megabase (Nakamura et al., 2015; Lin et al., 2021). A phase II study (JS001-ZS-BC001) of toripalimab (PD-1 inhibitor) plus S-1 and gemcitabine had promising survival benefits in untreated BTC patients with ORR of 27.1% and a median OS of 16 months (Li et al., 2021). Biomarker analysis revealed that TMB was not associated with treatment response or survival outcomes in JS001-ZS-BC001, which was further validated by the phase II study of camrelizumab (PD-1 inhibitor) plus gemcitabine and oxaliplatin in untreated BTC patients (Chen et al., 2020). The efficacy of pembrolizumab on PD-L1-positive and negative CCA was analyzed in the KEYNOTE-158 trial. PD-L1 positivity was not associated with superior survival outcomes, and there were no significant differences in median PFS (1.9 vs. 2.1 months) or OS (7.2 vs. 9.3 months) between PD-L1- positive and negative subgroups (Bang et al., 2019). In the subgroup of TOPAZ-1 trail, the addition of durvalumab to chemotherapy benefited patients with tumors characterized by a PD-L1 tumor area positivity (TAP) of 1% or greater and a TAP of less than 1%, suggesting that PD-L1 status may have limited value in predicting clinical benefit with chemoimmunotherapy in BTC (Oh et al., 2022). Additionally, trials of chemoimmunotherapy have been completed for patients with non-small cell lung cancer (NSCLC), even independent of PD-L1 status. In the KEYNOTE-189 and KEYNOTE-407 trials, NSCLC patients with less than 1% PD-L1 expression also responded to chemoimmunotherapy, and PD-L1 expression did not correlate with the clinical benefit (Gandhi et al., 2018; Yan et al., 2018). It is worth mentioning that the accuracy of these biomarkers is controversial. The biological process of anti-tumor immune response is complicated, involving cancer cells and cells in the TME. The different biomarkers accounted for few aspects of the overall process and, therefore, cannot be used to predict efficiently. For example, in this case, we comprehensively analyzed the genomic alterations and the immune microenvironment and found that our patient, with MSS, low TMB, negative CD8^+^ TIL and PD-L1 expression, can still benefit from chemoimmunotherapy, even in the second-line treatment. Further exploration of predictive biomarkers of response to immunotherapy for BTC is needed.

The optimal duration of ICI maintenance therapy remains controversial. Currently, the immunotherapy duration designed in most studies for advanced tumors is two years or discontinued when tumor progression or intolerable toxicities appear. However, several studies have shown that patients who were sensitive to immunotherapy but discontinued it due to immune-related adverse events had similar survival outcomes to those who completed the established therapy, indicating that early discontinuation of immunotherapy had no impact on survival (Schadendorf et al., 2017; Horiguchi et al., 2018; Kimura et al., 2019). In the current case, no significant change in tumor size was observed after switching to pembrolizumab monotherapy, but immune-related adverse effects occurred. Interestingly, no considerable tumor changes were observed even 14 months after discontinuing maintenance therapy. Although controllable CIP occurred on this patient, we will consider reintroducing nab-paclitaxel plus pembrolizumab if tumor progression in the following follow-ups because the patient has been off this treatment for more than one year.

## Conclusion

ICI combined with nab-paclitaxel might be a potential treatment option for patients with CCA and further clinical trials are needed to explore the outcomes.

## Data Availability

The original contributions presented in the study are included in the article, further inquiries can be directed to the corresponding author.
